# Does Fair Coach Behavior Predict the Quality of Athlete Leadership Among Belgian Volleyball and Basketball Players: The Vital Role of Team Identification and Task Cohesion

**DOI:** 10.3389/fpsyg.2021.645764

**Published:** 2022-02-07

**Authors:** Maarten De Backer, Stef Van Puyenbroeck, Katrien Fransen, Bart Reynders, Filip Boen, Florian Malisse, Gert Vande Broek

**Affiliations:** Physical Activity, Sports and Health Research Group, Department of Movement Sciences, Faculty of Movement and Rehabilitation Sciences, KU Leuven, Leuven, Belgium

**Keywords:** organizational justice, fairness, social identity approach, group dynamics, team sports, coaching

## Abstract

A vast stream of empirical work has revealed that coach and athlete leadership are important determinants of sport teams’ functioning and performance. Although coaches have a direct impact on individual and team outcomes, they should also strive to stimulate athletes to take up leadership roles in a qualitative manner. Yet, the relation between coach leadership behavior and the extent of high-quality athlete leadership within teams remains underexposed. Based on organizational justice theory and the social identity approach, the present research tested whether perceived justice of the coach positively predicts the quality of athlete leadership. Furthermore, we examined the role of group dynamic processes (i.e., team identification and task cohesion) within this relation. Belgian volleyball (*N* = 161) and basketball players (*N* = 78) were asked to rate the justice of their coach, their team identification, the task cohesion, and the athlete leadership quality in the team. Structural equation modeling indicated that coaches’ perceived justice positively predicted the quality of athletes’ leadership, and that this relation was established through three intermediate steps (i.e., from team identification to task cohesion, to athlete leadership quality). These results suggest that fair coach behavior does not only bridge the gap between leadership and followership, it also has the potential to improve the quality of athletes’ leadership within sport teams. More specifically, findings suggest that coaches’ perceived justice cultivates a shared social identity characterized by high levels of players’ identification with their team, which in turn increased their perceptions of the team’s task cohesion. Finally, this increased task cohesion encouraged the athlete leaders to demonstrate high-quality leadership.

## Introduction

Bringing talented players together is only the first step toward success in team sport competition. The second and more important step is persuading these players to function together as a team. It is this team functioning that often makes the difference between winning and losing. Effective leadership of the coach plays an important role in the process of optimizing this team functioning (Cotterill, 2013). Research in the business setting concluded that leadership effectiveness predicts optimal team functioning (Stoker et al., 2001; Judge et al., 2004) and depends on the perceived justice of the leader (Colquitt and Greenberg, 2003; van Knippenberg et al., 2007). In line with these results, research in the sport setting (De Backer et al., 2011, 2015) has shown that when coaches are perceived as fair, athletes would more strongly identify with their team. More specifically, instead of defining themselves in terms of their personal identity (as “I” and “me”), players would rather define themselves as members of their team (as “we” and “us”) and strongly valued this group membership. Moreover, it has been shown that a high level of team identification and team cohesion decreased the amount of social loafing within sport teams (De Backer et al., 2011, 2015). Recent research also indicated that athletes who perceived their coach as fair showed higher levels of satisfaction with the working method of their coach and reported more progression (De Backer et al., 2020).

However, the coach is not the only source of leadership that can influence the team functioning. Also leaders within the team can occupy important leadership roles. These athlete leaders have been defined as “athletes, occupying a formal or informal role within a team, who influence a group of team members to achieve a common goal” (Loughead et al., 2006). Recent work has demonstrated that these athlete leaders have the potential to improve their team’s functioning, performance, and teammates’ well-being (Mertens et al., 2018; Fransen et al., 2020c). As a result, it seems valuable to examine whether and how coaches can stimulate athletes to take up leadership roles in a qualitative manner.

The first studies on athlete leadership in sport teams distinguished between three different leadership roles (Loughead et al., 2006): (1) Task leadership, which focused on the accomplishment of the team goals on the field (e.g., offering teammates tactical instructions when required); (2) Social leadership, which fostered on cultivating positive interactions between team members outside the field (e.g., offering support to teammates and caring for a good atmosphere off the field); and (3) External leadership, aiming for a good representation of the team toward people outside the team, such as media, sponsors, …. However, research of Fransen et al. (2014b) demonstrated the existence of a fourth distinct role, namely the motivational leader on the field. This motivational leader encourages teammates to stay motivated during games and practices (e.g., by encouraging teammates to do their utmost on the field). Fransen et al. (2014a) demonstrated that each of the four leadership roles contributes to an overall perception of athlete leader quality. High-quality athlete leadership in the team has been linked to higher levels of team cohesion (Price and Weiss, 2011, 2013; Fransen et al., 2014b), team confidence (Fransen et al., 2014a), and even team performance (Fransen et al., 2015a).

It is thus well-known that qualitative athlete leaders positively impact the team’s functioning and several performance-enhancing outcomes. Yet, research on athlete leadership has almost exclusively focused on outcomes of high-quality leadership within teams, thereby ignoring how the quality of athlete leaders can be fostered within a team. Only recently, scholars have started to develop intervention protocols to develop athlete leadership (e.g., Fransen et al., 2020b). Yet even these studies mainly target players within the team, thereby underlighting the potential role of coaches’ leadership style and coach behavior in stimulating high-quality athlete leadership within their teams. The current study aimed to address this question by investigating the relation between the perceived justice of the coach and athlete leadership quality in sport teams. De Backer et al. (2011, 2015) already referred to the importance of the perceived justice of the coach for shaping team identification and cohesiveness within the team. We assume that such positive group dynamics are key conditions to foster high qualitative athlete leadership.

In order to gain insight in the process through which a fair coaching style could foster high qualitative athlete leadership, we draw on the organizational justice theory (Greenberg, 1987). This theory describes and explains the importance of a leader’s fairness in the workplace (Greenberg, 1990). Scientists have translated organizational justice to the team sport context (Jordan et al., 2004), in which they have focused on the three original subtypes of organizational justice: distributive justice (i.e., the perceived fairness of decision outcomes such as the playing time; Adams, 1965): procedural justice (i.e., the perceived fairness of the procedures used to obtain outcomes, such as the use of objective scouting information; Thibaut and Walker, 1975), and interactional justice (i.e., the interpersonal treatment and the information individuals receive from the coach; Bies and Moag, 1986).

Organizational justice research has been characterized by studies on the unique effects of these different types of justice. However, researchers have demonstrated that individual’s justice perceptions may not be accurately evaluated when the various dimensions of justice are differentiated (Hauenstein et al., 2001). Therefore, a shift toward examining overall justice judgments is recommended (Ambrose and Arnaud, 2005). For example, Törnblom and Vermunt (1999) stated that the components of fairness are only meaningful in relation to the overall fairness of the situation (i.e., the justice of a situation as a Gestalt). Accordingly, Greenberg (2001) suggested that when individuals form justice perceptions, they do so with a “holistic judgment in which they respond to whatever information is both available and salient” (p. 211). In line with these suggestions, the present research used the composite score of the three perceived justice subcomponents and aimed to study whether athletes’ overall perceived justice of the coach predict athletes’ leadership quality.

As mentioned before, to our knowledge no research has been performed on the effect of coach behavior on athletes’ leadership quality. However, previous research in the team sport setting clearly demonstrated that coaches’ behavior strongly predicts the extent to which team members’ take initiative by correcting others or providing suggestions for improvement (Van Puyenbroeck et al., 2017). In addition, research on justice in sport did support a positive link between the perception of fair coach behavior and athletes’ team identification and team cohesion (De Backer et al., 2011, 2015). Both team identification and cohesion are known to be crucial for group-oriented behavior, such as cooperative behavior, task performance, and the amount of effort that people are willing to exert for their team (Haslam, 2004; Høigaard et al., 2006). Furthermore, De Backer et al. (2015) showed that team identification and task cohesion mediate the relation between perceived fairness of the coach and athletes’ social loafing.

As a result, this research does not only aim to provide evidence that the perception of justice is an important antecedent of high-quality athlete leadership in sport teams. It also aims to explore the pathways that lead from the perception of justice to the perception of high-quality athlete leadership. Therefore, our hypothesized model was not only grounded on the organizational justice theory, but also on the social identity approach (SIA; Haslam, 2004). This theory, which distinguishes between a personal identity and a social identity, explains how the perceived fairness of the coach fosters athletes’ social identity, which in turn is positively linked to increased levels of task cohesion on the team. Personal identity refers to the self as a unique individual, while social identity refers to the self as an interchangeable group member (i.e., people’s sense of themselves as part of “us”). Furthermore, SIA states that perceiving the self as an interchangeable member of a category (i.e., the self-categorization process) is the cognitive process associated with social identity. Turner (1982) argued that the “switching on” of social identity is the cognitive mechanism that makes group behavior possible. Consequently, when an athlete identifies with the team (e.g., based on situational incentives such as the presence of an opponent team), this social identity will dominate, which in turn will lead to the internalization of the norms and behaviors prescribed by this group.

The closely related concept of team cohesion was defined by Carron et al. (1998, p. 213) as “a dynamic process that is reflected in the tendency for a group to stick together and remain united in the pursuit of its instrumental objectives and/or for the satisfaction of member affective needs.” They distinguished between a social subcomponent (i.e., a general orientation toward developing and maintaining social relationships within the group) and a task subcomponent (i.e., a general orientation toward achieving the group’s goals and objectives). Both components have been further differentiated into an individual (i.e., individual attraction to the group) and a group component (i.e., group integration). Previous research (Heuzé et al., 2006; De Backer et al., 2015) has indicated that especially task cohesion plays a vital role in the group functioning of elite sport teams.

The group engagement model (Tyler and Blader, 2003) connects both the organizational justice theory and the SIA. More precisely, it indicates that the impact of perceived justice on peoples’ engagement in groups is mediated by identity judgments. In support of the group engagement model, De Backer et al. (2011, 2015) showed that high perceived justice of the coach shapes high levels of identification with the team, which in turn increases the team cohesion and decreases the social loafing among team athletes. The group engagement model explains this increased team identification as a logical consequence of the fact that two essential functions of organizational justice (i.e., quality of decision making, and quality of interpersonal treatment) contribute to people’s assessment that it is safe for them to merge their identity with their group. Furthermore, the results of De Backer et al. (2011, 2015) showed that team identification and team cohesion were closely related but different constructs. In line with these results, Dutton et al. (1994) stated that the perception of a shared categorical identity (i.e., team identification) creates an in-group bias by accentuating the perceived similarities with other group members and results in positive attitudes toward these in-group members. This process eventually leads to increased intragroup cohesion (Dutton et al., 1994). In other words, team identity is the fundamental process of internalizing norms and values of a group, which lead to more process-based outcomes such as task cohesion.

Finally, in line with a previous statement of Zaccaro et al. (2001), we assume that the relation between leadership and team processes (i.e., team identification and cohesion) is not unidirectional, but bidirectional. More specifically, we expect that these team processes may also foster athletes’ leadership quality. Research in business settings has revealed that members of highly cohesive and more specifically task cohesive groups show more qualitative leadership behavior: (a) They plan more efficiently and develop more appropriate performance strategies (Hackman and Morris, 1975; Hackman, 1976); and (b) They set and enforce stringent performance norms to compel maximal effort of all team members (Zaccaro and McCoy, 1988). Zaccaro et al. (2001) confirmed that task-oriented cohesion is associated with strong work norms and that once these norms have been established, they are enforced by the members themselves (e.g., by communicating in various ways with non-conforming individuals to bring them in line with group work expectations). These behaviors closely align with athlete leadership behaviors. Therefore, we expect that task cohesion in particular will be positively related to athletes’ leadership quality.

To summarize, and based on this theoretical background, we expect that perceived fairness of the coach will positively predict athletes’ leadership quality. More specifically, we expect that this prediction will be established through three intermediate steps. That is, we expect that coaches’ perceived justice will foster athletes’ team identification, which in turn is hypothesized to positively predict task cohesion. The increased levels of task cohesion, in turn, are expected to be related to increased levels of perceived athlete leadership quality.

*Hypothesis 1: Athletes’ perceived justice of the coach positively predicts the perceived quality of athlete leadership.*

*Hypothesis 1a: Athletes’ perceived justice of the coach positively predicts team identification.*

*Hypothesis 1b: Team identification positively predicts task cohesion.*

*Hypothesis 1c: Task cohesion positively predicts athlete leadership quality.*

**FIGURE 1:**
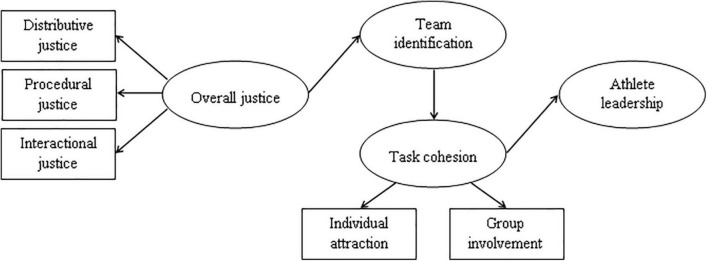
The hypothesized structural model of overall justice, team identification, task cohesion, and athlete leadership quality.

All hypotheses were combined into one comprehensive research model (Figure 1).

## Materials and Methods

### Participants and Procedures

#### Participants

We defined our sample size using the proposed ratio of sample size/parameters by Kline (2005). He argues that this ratio should at least be 5:1. Our model includes 30 parameters that need to be estimated, which requires at least a sample size of 150 athletes. Based on the response rates of previous studies in sport teams (e.g., Van Puyenbroeck et al., 2020), we contacted 30 teams in order to obtain this sample size. To recruit the research sample, we first listed all Flemish (i.e., Dutch speaking part of Belgium) basketball and volleyball clubs from the highest national to the first regional level of the Belgian competitions. Second, we randomly and blindly selected 18 Belgian volleyball and 12 Belgian basketball teams. Consequently, the head coaches of these 30 teams were contacted by telephone and informed about the purpose and the design of the research. Twenty-six coaches allowed their teams to take part in the study (i.e., seven male and 11 female volleyball teams, and five male and three female basketball teams). Four basketball coaches indicated that the workload of their players was too high and refused to participate in the study. The final research sample consisted of 239 senior athletes (i.e., 62 male and 99 female volleyball players, and 54 male and 24 female basketball players). This gives a total response rate of 81.3% (i.e., the response rate was respectively, 81.3% for volleyball, and 81.2% for basketball). It should be noted that the response rates for two volleyball teams were significantly lower (i.e., 36.4 and 45.5%) than the response rates of the other 24 teams (i.e., at least 58%). The lower response rates could be partly explained by the fact that both teams struggled with a lot of injured players. The mean age of the players was 23.10 years (*SD* = 4.95) and they had worked together for on average 2.17 years with their current coach (*SD* = 1.84).

#### Procedure

During or after a practice, we verbally informed the athletes about the objectives of our study and invited them to participate. The accurate timing of this briefing depended on the coach’s preference. Athletes who agreed to participate first provided their written informed consent and afterward completed a paper-and-pencil questionnaire. A trained research assistant was present to clarify ambiguities and answer possible questions. The current study was approved by the Doctoral School of Biomedical Sciences (i.e., by the Doctoral Committee of Kinesiology, Rehabilitation Sciences and Physiotherapy) of the KU Leuven. Furthermore, the ethical standards of the American Psychological Association (APA) were followed in the conduct of the study. No rewards were given for participation, and prior to completing the questionnaire, it was stated that participation was completely voluntary and that the players’ anonymity was guaranteed. Prior to the data analysis, the names of the athletes and teams were replaced by numeric athlete and team ID’s. The analyses were performed on this dataset. The original pencil-and-paper questionnaires were stored in a locked cupboard, thereby complying to the research institute’s data management regulations. No individual or team scores were shared with the coaches or other athletes/teams. We emphasized the importance of responding independently and honestly to the questions.

### Measures

#### Perceived Justice (Nine Items)

Justice perceptions were assessed with nine items selected from a 12-item justice measurement used in previous research (De Backer et al., 2015). This Dutch justice measurement was based on the justice questionnaire developed by Colquitt (2001) in the business setting and used a 5-point Likert scale (*strongly disagree* = 1; *strongly agree* = 5). We shortened the questionnaire to limit the workload of the athletes, by selecting the three highest loading items of each of the three subscales previously used in the team sport setting by De Backer et al. (2015). The shortened nine-item measurement consisted of three items that assessed the perception of distributive justice (e.g., “The minutes I play per game are a true reflection of my commitment and efficiency during the game”), three items that assessed the perception of procedural justice (e.g., “The decisions of my coach are based on objective information”), and three items that assessed the perception of interactional justice by evaluating the degree to which the procedures and outcomes are clear for the athletes (e.g., “My coach motivated and argued his decisions”). A second-order confirmatory factor analyses (CFA) established that the nine items formed three subcategories of justice (distributive, procedural, and interactional justice), which in turn significantly contributed to an overall measurement of perceived justice (χ^2^ = 46.61, *df* = 23, *p* = 0.00; χ^2^/df = 2.03; GFI = 0.96; CFI = 0.98; RMSEA = 0.07). The internal consistency of the overall justice scale (Cronbach’s α = 0.87) was high.

#### Identification With the Team (Five Items)

Team identification was measured using five items. These items were based on the fan identification scale constructed by Boen et al. (2008). We slightly rephrased the items to fit the specific team sport context (e.g., “this team” replaced “my old club”). The reliability of this adapted scale was already demonstrated in a sample of Flemish team athletes (Fransen et al., 2014a, 2015a; De Backer et al., 2015). The five items used a 7-point Likert scale (*strongly disagree* = −3; *strongly agree* = 3). An example item is “I strongly identify with this team.” CFA showed good fit to the data (χ^2^ = 3.05, *df* = 3, *p* = 0.38; χ^2^/df = 1.02; GFI = 1.00; CFI = 1.00; RMSEA = 0.01) and the internal consistency of the five-item scale was excellent (Cronbach’s α = 0.91).

#### Task Cohesion (Nine Items)

Task cohesion was questioned with the two task-related subcomponents of the Group Environment Questionnaire (GEQ; Carron et al., 1998) using a 9-point Likert scale (*strongly disagree* = *1; strongly agree* = *9*). Four items assessed the individual attraction to the group-task subcomponent (e.g., “I am unhappy with the team’s level of desire to win”), and five items assessed the group involvement-task subcomponent (e.g., “Our team is united in trying to reach its performance goals”). The CFA of the two-factor task cohesion measurement showed an acceptable fit to the data (χ^2^ = 63.91, *df* = 24, *p* = 0.00; χ^2^/*df* = 2.66; GFI = 0.95; CFI = 0.96; RMSEA = 0.08). However, the factor loading for one item of the individual attraction to the group subcomponent (i.e., “I am satisfied with the playing time I get”) was low (0.24). When this item was removed, the fit of the model improved significantly (χ^2^ = 26.86, *df* = 17, *p* = 0.06; χ^2^/*df* = 1.58; GFI = 0.97; CFI = 0.99; RMSEA = 0.05) and the internal consistency of the individual attraction to the group subcomponent increased from α = 0.72 to α = 0.80. Therefore, we removed this item for further analyses.

Another consideration was the high correlation (*r* = 0.88) between the two task cohesion subcomponents in this two-factor model. In addition to this high correlation, the Cronbach’s α for a combined subscale of overall task cohesion (0.89) was higher than the Cronbach’s α for individual attraction to the group-task (0.80) and group involvement-task (0.84) separately. Therefore, we decided to combine the individual attraction to the group-task (three items) and the group involvement-task (five items) subcomponents into one latent variable (i.e., overall task cohesion) for the following main analyses.

#### Athlete Leadership Quality (Four Items)

In line with previous overall leadership research (Chemers et al., 2000; Fransen et al., 2014a) we opted for a single-item approach of athletes’ leadership quality. Tenenbaum et al. (2007) and Tenenbaum and Gershgoren (2011) already argued for a higher ecological validity of such single-item measurements. The current study examined the overall perceived leadership quality of the four athlete leaders on each of the leadership roles (i.e., task, motivational, social, and external leader). First, the exact descriptions of the four leadership roles, as outlined in previous research (Fransen et al., 2014b) and displayed in Supplementary Appendix 1, were presented to the participants. With these descriptions in mind, players had to appoint the player in their team who corresponded best to the description of the four leaders. Subsequently, participants were asked to complete the item “To what extent do you think that this leader fulfils his/her role as task/motivational/social/external leader well?” for each of the appointed leaders on a 7-point Likert scale (*very bad* = −3; *very good* = 3). This measurement already showed to be reliable and valid in a sample of Flemish team sport athletes (Fransen et al., 2014b). CFA confirmed that each of the four different leadership roles significantly contributed to an overall measure of perceived athlete leader quality (χ^2^ = 0.85, *df* = 2, *p* = 0.66; χ^2^/*df* = 0.42; GFI = 1.00; CFI = 1.00; RMSEA < 0.001).

### Data Analysis

First, the hypothesized model was examined through Structural Equation Modeling (SEM) with Mplus (Muthén and Muthén, 2017). Mplus also allows us to control for the nested structure of our data, as players were nested within teams, by using the TYPE = complex command. If we would ignore this nested structure and only test a simple single-level model using SEM, the standard errors would be inflated resulting in Type I error. The statistical procedure used in this study therefore adjusts the standard errors to prevent them from being inflated due to clustering (for more information, see McNeish et al., 2017; Muthén and Muthén, 2017).

The skewness of the studied variables ranged from −1.56 to −0.05, which are considered acceptable values when conducting SEM (Brown, 2015). SEM is a robust analytical technique of which the assumptions are not sensitive to such small deviations (Griffin and Steinbrecher, 2013). Furthermore, we used the robust maximum likelihood estimator (MLR) for the estimation of our models, which is robust to non-normality and non-independence of observations when used with TYPE = complex command (Muthén and Muthén, 2017).

We used the following fit indices to evaluate model fit: the normed chi-square statistic (χ^2^/df), the Comparative Fit index (CFI), the Tucker-Lewis index (TLI), and the Root Mean Square Error of Approximation (RMSEA). While a non-significant chi-square (χ^2^) implies a good fit of the model, the significance of this statistic is largely dependent on sample size. Accordingly, we used the normed chi-square statistic (χ^2^/df), where a good fit is reflected by a ratio below 3/1 (Kline, 2005). Furthermore, a good fit of the model to the data is signified by CFI and TLI values larger than 0.90 and an RMSEA equal or smaller than 0.08 (Hu and Bentler, 1999).

Finally, we tested an additional model in which we added a direct link between athletes’ perceived justice and athlete leadership quality. If this direct link is non-significant, in combination with a significant indirect effect of athletes’ perceived justice on athlete leadership in the hypothesized model, this would confirm that athletes’ perceived justice predicts athlete leadership quality through the expected intermediate steps.

## Results

### Descriptive Statistics, Correlations, and Scale Reliabilities

Scales, means, standard deviations, and correlations for the variables are provided in Table 1. Scale reliabilities (Cronbach’s alphas) are provided on the diagonal. The significant correlation (*r* = 0.21, *p* < 0.01) between athletes’ perceived justice of the coach and the quality of athletes’ leadership supports Hypothesis 1. Furthermore, we performed an ANOVA to check for differences between the volleyball and the basketball players. We found only one significant, and small difference of 0.24 in the mean score on the 7-point Likert scale for team identification. Taking into account that this was the only difference between both sports, we decided not to split the sample and to perform our main analyses on the combined research sample.

**TABLE 1:** Scales, means, standard deviations correlations, and Cronbach’s alphas for all variables.

	Variable	Scale	*M*	SD	1	2	3	4
1.	Overall justice	1, 5	3.46	0.81	*(0.87)*			
2.	Team identification	−3, 3	2.01	0.87	0.29**	*(0.91)*		
3.	Task cohesion	1, 9	6.57	1.29	0.45**	0.57**	*(0.89)*	
4.	Athlete leadership quality	−3, 3	1.53	0.74	0.21*	0.35**	0.30**	*(0.65)*

*p < 0.01; **p < 0.001. Scale reliabilities (Cronbach’s alphas) are provided in italics on the diagonal.

### Structural Equation Modeling

**FIGURE 2:**
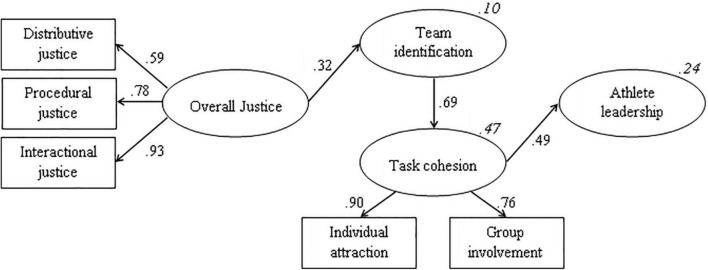
The structural model of overall justice, team identification, task cohesion, and athlete leadership quality with the regression coefficients and the proportions explained variance. All coefficients presented are standardized and significant (*p* ≤ 0.001).

The hypothesized model showed a good fit to the data (χ^2^ = 146.25, *df* = 74, *p* < 0.001; χ^2^*/df* = 1.98; TLI = 0.93; CFI = 0.94; RMSEA = 0.06). The standardized regression path coefficients and the proportions explained variance are illustrated in Figure 2. The results demonstrated that athletes’ perceived fairness of the coach positively predicted team identification. Team identification positively predicted task cohesion which, in turn, positively predicted athlete leadership quality. In addition to the effects that are presented in Figure 2, all standardized indirect effects of the hypothesized model are depicted in Table 2. Further, we added a direct link between athletes’ perceived fairness of the coach and athlete leadership quality. This direct link was non-significant (β = 0.18, *p* = 0.09), while the indirect effect of athletes’ perceived justice on athlete leadership quality, through team identification and, in turn task cohesion, was significant within the hypothesized model (β = 0.11, *p* = 0.01). The results of this analysis confirmed that athletes’ perceived justice predicts athlete leadership quality through the expected intermediate steps.

**TABLE 2:** Standardized indirect effects and standard errors (SE) for all paths in the model between predictors (in rows) and outcomes (in columns).

	Task cohesion	Athlete leadership quality
	*Effect (SE)*	*Effect (SE)*
Overall justice	0.22** (0.06)	0.11* (0.04)
Team identification		0.34** (0.08)

*p < 0.05; **p < 0.001.

## Discussion

Recent research indicated that high-quality athlete leaders improve the effective functioning of sport teams. More specifically, some studies have demonstrated the positive link between athlete leaders and team functioning and performance in sport teams (Fransen et al., 2015a; Mertens et al., 2018). Despite these promising results, no research has examined the role of coaches’ behavior in fostering the development of the athlete leadership quality in team sports. The current research demonstrated that a specific aspect of coach leadership, namely coaches’ fairness, was positively related to athletes’ leadership quality through its inter-relations with athletes’ team identification and task cohesion, thereby confirming our hypotheses.

First, when coaches were perceived as fair, the identification of the athletes with their team seemed stronger. A possible explanation for this positive relation can be found in the group engagement model (Tyler and Blader, 2003). This group engagement model assumes that “perceived justice provides key information that shapes the degree to which people regard their group as having high status, regard themselves as having high status in their group, and identify with the group by merging their sense of self with the group” (Tyler and Blader, 2003, p. 357). These results are in line with the statement of Haslam et al. (2011) that leadership is an active process that has the ability to shape social identities. Furthermore, our findings support the theoretically based assumption that team identification is a fundamental process of internalizing norms and values of a group, which ultimately leads to more intragroup cohesion. In other words, our results are in line with the social identity mediation hypothesis, which suggests that identity evaluations and concerns mediate the relationship between justice judgments and group engagement (Tyler and Blader, 2003).

Second, the current research indicated that fair coach behavior positively predicted athletes’ leadership quality and that this prediction was established through three intermediate steps. That is, coaches’ perceived justice fostered athletes’ team identification, which in turn positively predicted task cohesion. The increased levels of task cohesion, in turn, were related to increased levels of perceived athlete leadership quality. These results suggest that fair coach behavior does not only bridge the gap between leadership and followership (Haslam et al., 2011), it also has the potential to improve the quality of athletes’ leadership within sport teams. More specifically, fair coach behavior seems to guide the important group processes of team identification and task cohesion, and as a result shapes a climate in which athletes get the opportunity to develop qualitative leadership. A possible explanation for the predictive value of justice for athletes’ leadership quality can be found in the statement of Haslam et al. (2011, p. 110–111) that “leader’s fairness can unite us by both creating and clarifying shared group memberships, and in this way, that it can become a basis for influence and inspirational leadership.” Indeed inspirational leadership is known to: (a) Reinforce the common goals of the team (i.e., task cohesion) and (b) Encourage interpersonal interaction among team members (Joshi et al., 2009).

Setting clear and common goals as well as high-quality interpersonal interactions are essential conditions for qualitative leadership behaviors in sport teams. Furthermore, previous research (Hackman and Morris, 1975; Hackman, 1976; Zaccaro and McCoy, 1988) demonstrated that members of highly task cohesive groups: (a) Plan and develop efficient and appropriate performance strategies (i.e., task leadership), and (b) Compel maximal effort of all team members by setting and enforcing stringent performance norms (i.e., motivational leadership). In addition, Zaccaro et al. (2001) indicated that team members with a high perception of task cohesion communicate in various ways with non-conforming individuals to bring them in line with group work expectations (task and motivational leadership). In line with those findings, our results suggested that team identification and task cohesion are intermediate steps in the relation between perceived justice of the coach’s and athletes’ leadership quality.

### Limitations and Practical Implications

As with any research, the current study had not only strengths, but also specific limitations. A first limitation is the cross-sectional nature of our data, thereby limiting our ability to infer causality from the results. Based on previous research (Hackman and Morris, 1975; Hackman, 1976; Zaccaro and McCoy, 1988; Zaccaro et al., 2001; Haslam et al., 2011), we constructed a theoretically founded research model. In line with those studies, our results supported the fact that group dynamical processes (i.e., team identification and task cohesion) form the intermediate steps in the relation between coaches’ justice and athletes’ leadership quality. Nevertheless, some previous research also indicated that athlete leadership qualities positively predict athletes’ team identification and cohesion in sport teams (Fransen et al., 2014a). While our results seem contradictory to these previous findings, Zaccaro et al. (2001) indicated that the relation between leadership and team processes is reciprocal and not unidirectional (i.e., leadership and team processes influence each other). As a result, longitudinal and experimental studies are required to assess the direction of the different relations and to explore how these relations fluctuate across a season.

Another reason to be cautious when interpreting the significance of our findings is the lack of control variables or other potential predicting variables in the model. For example, previous studies revealed the importance of a mastery-oriented climate in predicting the extent of initiative and constructive peer corrections within sport teams (Van Puyenbroeck et al., 2017). Others demonstrated that specific behaviors of the athlete leaders (e.g., problem-solving skills) or certain personality traits (e.g., extraversion) also predict the quality of athlete leaders (Fransen et al., 2020a). Future work should therefore include more variables as control variables or as additional potential mechanisms that predict athlete leadership in addition to this study’s variables. When these relations would be confirmed, this would increase the validity of our study findings and the significance of its implications.

Second, we assessed leadership quality with a commonly used measurement of athlete leadership quality. This one-item measure assessing the perceived quality with which athlete leaders fulfilled their specific leadership role showed to be a valid measure both in previous studies (Fransen et al., 2014a) and in the current study. In this study, we asked participants to rate the quality of the best leader in their team (on the different leadership roles). However, only rarely leadership is occupied by only a single team member. Previous studies have shown that leadership is rather shared, not only across, but also within each of these leadership roles (e.g., Leo et al., 2019). Therefore, future studies should consider using a social network approach, in which the leadership quality of every team member is assessed, rather than only of the best leader (e.g., Fransen et al., 2015b).

Third, although we controlled for the nested structure of our data, we did not conduct a multilevel SEM with a second level that included all of our variables at team level as our sample consisted of players from only 26 different teams. Maas and Hox (2005) stated that it is not recommended to perform such multilevel analyses based on such a small sample size at team level (i.e., level 2). However, the variables of interest (e.g., perceived justice, team identification, task cohesion, and athletes’ leadership qualities) potentially exhibit a significant degree of intra-group consensus within sport teams. In this study, the within-group agreement (r_wg(j)_; James et al., 1984) was moderate to high for perceived justice (r_wg(j)_ = 0.82), team identification (r_wg(j)_ = 0.91), task cohesion (r_wg(j)_ = 0.65), and athlete leadership quality (r_wg(j)_ = 0.91). For this reason, future research should sample a larger number of teams and simultaneously test the hypothesized relations at team level.

Notwithstanding those limitations, we want to underline that the current study was an important first step in the examination of the link between perceived fair coach behavior and athletes’ leadership quality. More specifically, the interrelations between perceived justice, team identification (SIA), task cohesion, and athletes’ leadership qualities offer important insights into the mechanisms that underpin the impact of coaches’ justice on the development of qualitative leadership behavior of senior team athletes.

From a more practical point of view, our comprehensive research model indicates that the perceived fairness of team coaches may possibly affect key group processes and consequently foster the quality of athlete leadership. Previous research has shown that high-quality athlete leaders improve the effective functioning of sport teams (Price and Weiss, 2011, 2013; Fransen et al., 2014a, 2015a). As a result, our model can be used to optimize team performance in senior interactive sport teams.

An important practical take-away of our study is the fact that if we value high-quality athlete leaders, we must not lose sight of the impact of coach behavior. Nowadays, athlete-oriented leadership development programs receive a lot of attention. However, our results indicate that the quality of athlete leadership is not only the result of specific leadership development programs that target team athletes. It is also related to specific group dynamical processes driven by fair coach behavior.

Therefore, coaches should be aware of the importance of how athletes perceive their justice. Research in the business setting suggested that there are a number of strategies, such as the application of Leventhal’s rules (Leventhal, 1980; e.g., be consistent, suppress bias, …), and the provision of voice (Skarlicki and Latham, 1996, 1997), to improve employees’ perception of fairness (Cropanzano and Greenberg, 1997). Both strategies have been shown to be effective even when people were disappointed with the outcomes they received. How leaders can apply these strategies is described in detail by multiple researchers within the business context (Leventhal, 1980; Skarlicki and Latham, 1996, 1997; Greenberg and Lind, 2000). For a more in-depth description of the application of organizational justice in a team sport setting we would like to refer to Jordan et al. (2004).

Furthermore, our findings highlight the team dynamics that underpin the relationship between fair coach behavior and the quality of athlete leadership. In this regard, we suggest that coaches of interactive sport teams should pay sufficient attention to create a sense of shared social identity that results in the integration of the individual tasks and goals of the players into the overall team objectives. For a more detailed overview of how leaders can create, represent, advance, and embed this sense of shared social identity, we would like to refer to Haslam et al. (2011). A practical example of how coaches could highlight that necessity of a sense of “us-ness” is by emphasizing that the team goals prevail over the individual goals at all times. Important in this process is the framing of an effective goal agreement, including commonly agreed goals for both the individual players and the team as a whole. This ensures that each player knows how every specific task fits within the bigger framework of the team. Consequently, we assume that a collectively agreed goal arrangement, due to the shared knowledge of the different tasks, will enhance athletes’ task cohesion and thus the quality of athletes’ leadership.

To conclude, this study supported a positive link between the perceived fairness of team coaches and athletes’ team identification and task cohesion. This increased team identification and task cohesion in turn leads to increased perceived athlete leadership quality. Based on the current findings, the organizational justice theory seems to be a promising theoretical framework to underpin the impact of coaches’ leadership in sport settings. From a practical point of view, fair coaches strengthen the quality of athletes’ leadership and potentially may lead to a more optimal team functioning. Therefore, coaches should not only attempt to act in a fair manner toward all team members but should also make sure that their actions are interpreted as fair by team members.

## Data Availability Statement

The raw data supporting the conclusions of this article will be made available by the authors, without undue reservation.

## Ethics Statement

The studies involving human participants were reviewed and approved by the Leuven International Doctoral School Biomedical Sciences. The Doctoral Committee of Kinesiology, Rehabilitation Sciences and Physiotherapy. The patients/participants provided their written informed consent to participate in this study.

## Author Contributions

MD, KF, FB, and GV contributed to the design of the research. MD, SV, KF, BR, FB, and GV contributed to the implementation of the research. MD, SV, KF, BR, FB, FM, and GV contributed to the analysis of the results, and writing of the manuscript. All authors contributed to the article and approved the submitted version.

## Conflict of Interest

The authors declare that the research was conducted in the absence of any commercial or financial relationships that could be construed as a potential conflict of interest.

## Publisher’s Note

All claims expressed in this article are solely those of the authors and do not necessarily represent those of their affiliated organizations, or those of the publisher, the editors and the reviewers. Any product that may be evaluated in this article, or claim that may be made by its manufacturer, is not guaranteed or endorsed by the publisher.
