# Supplementary material for: Does Fair Coach Behavior Predict the Quality of Athlete Leadership Among Belgian Volleyball and Basketball Players: The Vital Role of Team Identification and Task Cohesion
Source: Front Psychol. 2022 Feb 7;12:645764. doi: 10.3389/fpsyg.2021.645764 (PMC8858847; doi:10.3389/fpsyg.2021.645764)
Supplement: Supplementary file 1 [file Table_1.DOCX]

**Appendix 1**

Table 1A

The definitions of the four leadership roles, as outlined by Fransen and colleagues (2014).

| Leadership role | Definition |
| --- | --- |
| Task leader | A task leader is in charge on the field; this person helps the team to focus on our goals and helps in tactical decision-making. Furthermore the task leader gives his/her teammates tactical advice during the game and adjusts them if necessary. |
| Motivational leader | The motivational leader is the biggest motivator on the field; this person can encourage his/her teammates to go to any extreme; this leader also puts fresh heart into players who are discouraged. In short, this leader steers all the emotions on the field in the right direction in order to perform optimally as a team. |
| Social leader | The social leader has a leading role besides the field; this person promotes good relations within the team and cares for a good team atmosphere, e.g. in the dressing room, in the cafeteria or on social team activities. Furthermore, this leader helps to deal with conflicts between teammates besides the field. He/She is a good listener and is trusted by his/her teammates. |
| External leader | The external leader is the link between our team and the people outside; this leader is the representative of our team towards the club management. If communication is needed with media or sponsors, this person will take the lead. This leader will also communicate the guidelines of the club management to the team regarding club activities for sponsoring. |
